# Stigma, social appearance anxiety and coping in men and women living with skin conditions: A mixed methods analysis

**DOI:** 10.1002/ski2.73

**Published:** 2021-11-15

**Authors:** O. Hughes, P. B. Hutchings, C. Phelps

**Affiliations:** ^1^ School of Psychology University of Wales Trinity Saint David Carmarthen UK

## Abstract

**Background:**

The psychological impact of living with a skin condition can have a profound impact on quality of life and could cause appearance‐related social anxiety. Existing research suggests ambiguous findings in relation to whether the impact of living with a skin condition differs between males and females.

**Objectives:**

The present study aimed to explore the association between stigma, coping styles and social appearance anxiety in men and women living with a skin condition in the United Kingdom.

**Methods:**

231 participants (*n* = 199 females, *n* = 30 males, *n* = 2 non‐binary) completed a cross‐sectional online questionnaire, capturing quantitative data with the social appearance anxiety scale (SAAS), the shortened version of the coping inventory for stressful situations (CISS‐21), and qualitative data from free‐text comments and thematic content analysis. Respondents were also asked to provide additional free text comments in relation to the challenges faced and how these were managed.

**Results:**

Content analysis revealed that males and females faced daily practical, social and emotional challenges and coped with them in several ways; with higher levels of social appearance anxiety associated with both higher perceived severity of skin condition and younger age. Males and females appeared equally as emotionally affected by living with a skin condition, with the only significant gender difference being females as significantly more likely to engage in avoidant coping behaviours than males.

**Conclusions:**

Living with a skin condition presents daily practical, social, and psychological challenges for males and females that have the potential to impact on quality of life. Findings highlight the need for dermatological care to routinely address these issues, and psychosocial interventions must be made available to promote healthy coping with skin conditions.

## Graphical abstract

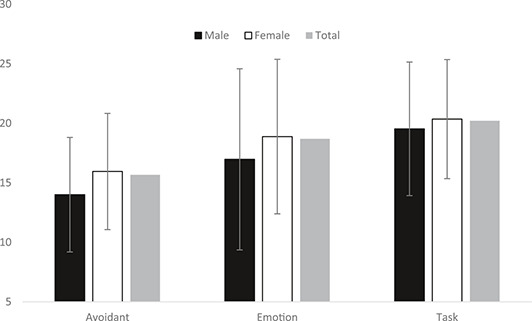

1

**WHAT'S ALREADY KNOWN ABOUT THIS TOPIC?**

- People living with skin conditions not only have to adapt to the physical discomfort associated with their diagnosis but may also experience a significant psychological impact ranging from lack of confidence, low‐self‐esteem, negative core beliefs about the self/appearance and, in some cases, clinical levels of depression, anxiety, and distress.

**WHAT DOES THIS STUDY ADD?**

- In line with stress and coping theory, individuals living with a skin condition appear to develop a range of coping strategies to help them navigate the ongoing practical and social challenges of their daily lives.
- Regardless of gender, there is an apparent need for greater recognition in routine care of the stigma, social anxiety, and challenges faced by people with skin conditions.

**WHAT ARE THE CLINICAL IMPLICATIONS OF THIS WORK?**

- Findings provide insights into the burden of living with a skin condition and importantly and illustrate how males and females appear to be equally psychosocially impacted.
- There is a need for more collaboration between clinical specialists and mental health professionals to embed an all‐encompassing treatment programme into the stepped care pathway for dermatology patients.

## 2 INTRODUCTION

There is growing recognition of the psychological impact of living with a skin condition and appearance‐related anxiety in a culture that still endorses unblemished appearance as ‘beauty’.^1^, ^2^ Individuals with skin conditions not only have to adapt to living with physical discomfort from their diagnosis but must also adjust psychologically to the associated psychological impact of not being able to meet unrealistic image ideals, which could range from lack of confidence, low‐self‐esteem and negative self‐beliefs, to comorbid mental health conditions^3^, ^4^, ^5^, ^6^, ^7^ such as depression and anxiety.^8^, ^9^

Stigmatisation from having imperfect skin could result in people limiting their life experiences through feeling self‐conscious about their appearance,^10^ and encountering challenges from finding ways of adjusting to living with a chronic illness combined with managing potentially negative reactions from others. Dealing with unwanted attention such as staring, intrusive comments, or even bullying could cause feelings of embarrassment and shame that can have a wider impact on inhibiting the formation of romantic relationships.^11^, ^12^ Individuals with skin conditions may also experience disruption to employment from attending appointments, time consuming treatments, and living with painful symptoms limiting earning potential.^10^, ^13^ These potentially negative outcomes could cause significant psychological stress, and ultimately cyclically interfere with the pathogenic mechanisms of the body's neuro‐immuno‐cutaneous endocrine system that precipitates flare‐ups in many skin conditions.^14^

Although there is some suggestion that individuals may develop effective coping strategies over time as they learn to adjust to a chronic skin condition,^15^, ^16^, ^17^ this could imply that younger people may be more psychologically affected.^18^, ^19^ In some cases, people may develop adaptive strategies to cope with their skin condition, but others may exhibit maladaptive or avoidant coping.^15^, ^20^ For example, withdrawing from social situations, becoming isolated, or experiencing a ‘self‐fulfilling prophecy’ from engaging in behaviours to conceal the skin and inadvertently attracting more attention (e.g., wearing unsuitable clothes for the weather).^15^ Despite the clear psychological sequelae of living with a dermatological condition, a recent survey by the All‐Parliamentary Group on Skin^21^ reported that 98% of patients surveyed felt their skin condition had affected their psychological wellbeing, only 18% had received psychological support, and over 5% of had experienced suicidal thoughts related to the impact of their skin condition.

The extent to which the psychological impact of living with a skin condition differs between males and females is unclear. It has been previously reported that females with atopic dermatitis and chronic dermatoses may experience greater depression and anxiety symptoms,^8^, ^22^, ^23^ higher stigma, and increased levels of social appearance anxiety than males.^3^ Further, females with skin conditions have been suggested to experience comorbid psychiatric conditions and suicide ideation more frequently than males.^24^, ^25^ However, findings regarding gender differences have been mixed, as other research has suggested little difference between levels of depression and anxiety in males and females with acne.^26^ It could be that the way in which the psychological impact manifests itself differs, with women being more likely to report depression and engage in emotion and/or avoidant behaviours, potentially mediated by romantic relationships, social support, levels of optimism, and self‐esteem.^17^, ^27^, ^28^, ^29^, ^30^

Given the complex challenges faced by people with skin conditions, and the mixed findings between males and females, the aim of this study was to explore whether levels of social appearance anxiety and types of coping strategies differ between males and females. Exploring the potential differences between how females and males may cope is vital for intervention development and for the design of targeted support resources for people with skin conditions, as first, any variations in precedence for target users must be identified. In recognition of the diversity of challenges and coping strategies that may exist, a mixed methods approach was adopted to capture quantitative and qualitative data to provide an in‐depth understanding of the impact of living with a skin condition, and to identify useful avenues for future interventions to improve coping and adjustment.

## 3 PATIENTS AND METHODS

### 3.1 Design

A UK‐wide online cross‐sectional questionnaire was employed, collecting quantitative data with two validated scales, demographic information, and qualitative data through four focused questions linked to free text boxes. Mixed methods approaches are recognised as being particularly valuable in healthcare research involving complex systems where clinical, societal, and psychological factors interact in complex ways, and the use of online surveys to collect such data is considered a pragmatic approach to capturing a wide sample of participants.^31^, ^32^

### 3.2 Participants

The target sample for the study were male and female adults (aged over 18) across the UK who identified themselves as having a skin condition. Participants were recruited through an online advertisement, asking for adults to participate in a survey exploring the psychological impact of living with a skin condition. The study advert was posted on social media and shared by relevant skin‐related charities and organisations.

### 3.3 Measures

Demographic data was collected for age (in years), gender, and type of skin condition (respondents could select multiple answers) from a list derived from the literature (psoriasis, eczema/dermatitis, acne, melanoma, port wine stain, vitiligo, rosacea, and ‘other’ free text). Current self‐reported severity (mild, moderate, or severe) was assessed by asking respondents to indicate ‘how severe do you believe your skin condition is?’ For the following two validated scales, respondents were asked to respond in relation their ‘skin condition’ in appropriate items to ensure responses were focused on the impact of living with a skin condition specifically.

The Social Appearance Anxiety Scale (SAAS)^33^ is a 16‐item self‐report inventory which measures social anxiety around situations whereby appearance may be evaluated. Responses are scored on a 5‐point Likert scale ranging from ‘not at all’ to ‘extremely’. The SAAS has high levels of test‐retest reliability, suggesting that levels of social appearance anxiety are stable across time.^33^, ^34^ Higher scores obtained from the scale indicate higher levels of social appearance anxiety, with possible scores ranging from 16 to 80. In the present study, the SAAS had a Cronbach's alpha of 0.96, indicating good internal consistency.

The shortened 21‐item Coping Inventory for Stressful Situations (CISS‐21)^35^, ^36^ is a self‐report inventory assessing three coping strategies: task‐orientated, emotion‐focussed and avoidance coping strategies, with responses scored on a 5‐point Likert scale ranging from 1 = ‘none at all’ to 5 = ‘extremely’. This scale is easily administered and has been shown to have strong psychometric properties.^37^ Scores for each sub‐scale are added up to create one overall score for each coping style dimension, with higher scores showing greater use of coping behaviours (total possible score for each sub‐scale 7 to 35). Cronbach's alphas for the subscales of the CISS‐21 scale for this study were 0.73 for avoidance coping, 0.88 for emotion‐focussed coping, and 0.79 for task‐orientated coping, indicating good internal reliability.

Qualitative data was collected using the following four open ended questions at the end of the online survey. First, ‘*please state the three main challenges you have found in living with a skin condition’*, second, *‘please could you describe the ways in which you manage these challenges?’*, third, ‘*how do you feel you could be better* *supported* *in living with your skin condition?’* and the final question asked participants if there was *‘anything else relating to coping with the psychological impact of your skin condition that you would like to mention?’*

### 3.4 Procedure

Ethical approval was obtained from the School Research Ethics Committee. Individuals interesting in taking part responded to the online advertisement by clicking on a link which took them directly to a webpage presenting the online questionnaire. Participants were asked to read the online study information sheet, provide their consent, and confirm their eligibility of having a skin condition and being over 18 years with a tick box. At the end of the survey participants were taken to a debrief page, which explained the aims and objectives of the questionnaire, thanked them, and provided contact details of the researchers.

## 4 RESULTS

### 4.1 Sample characteristics

A total of 231 participants responded (range 19–78 years) (*M* = 36.7, *SD* = 14.1). The following analyses included 229 complete data sets consisting of 30 males (13%), 199 females (87%). Data from two participants identifying as gender non‐binary were included in the overall analyses but excluded from the primary analyses and the qualitative content analysis relating to differences between males and females.

The most frequently reported skin condition was psoriasis (64%), followed by eczema/dermatitis (55.8%), acne (16%), rosacea (5.6%), vitiligo (1.3%), port wine stain (0.4%), melanoma (0.4%), and lastly, ‘other’ (not listed skin conditions) (6.1%). Some participants reported having more than one skin condition. The most commonly self‐reported severity of condition was ‘moderate’ (47.6%), followed by ‘mild’ (29.9%) and ‘severe’ (22.5%). The mean age of male respondents was 37.9 years (*SD = *16.8) and females, 36.6 years (*SD = *13.7). There was no significant association between gender and self‐reported perceived skin severity *x* ^2^(2, *n* = 229) = 0.84, *p* = 0.66, Cramer's *v* = 0.06.

**FIGURE 1:**
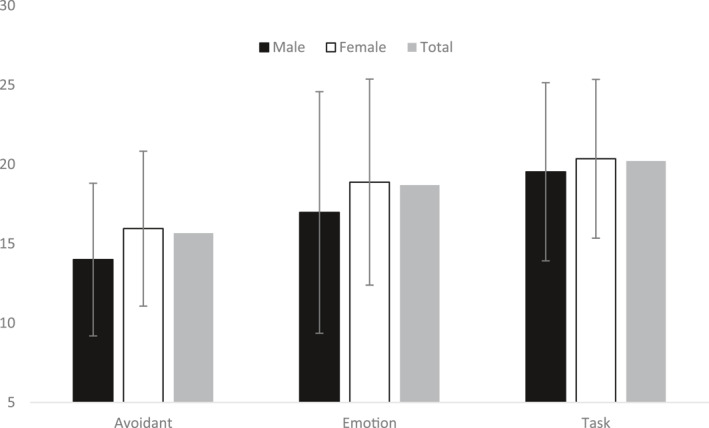
Mean scores as a function of coping style. Note: male and female scores on avoidant coping style; emotion‐focused coping style; and task‐focused coping style. Error bars denote standard deviation

Figure 1 shows the mean and standard deviation of avoidant, emotion‐focussed and task‐orientated coping scores for the total sample and also between males and females. A within‐subjects analysis of variance identified a significant difference in the extent to which participants made use of each of the three coping strategies (*F* (2, 229) = 71.93, *p* < 0.001). Post hoc tests revealed a significant difference in the frequency of use across all three coping strategies: the most frequently used form of coping was task‐oriented coping, followed by emotion‐focused coping, with avoidant coping strategies being used the least.

The only gender difference in use of coping strategies identified through a series of independent *t*‐tests revealed that females engaged in significantly higher levels of avoidance coping than males (*t* = −2.04, *p* = 0.04; 95%, CI [−3.83, −0.07]). There were no differences in level of engagement between males and females in either emotion‐focused coping or task‐oriented coping (see Figure 1). An additional independent *t*‐test also found no significant difference between males (*M* = 42.47, *SD* = 16.53) and females (*M* = 45.30, *SD* = 16.62) in social appearance related anxiety (*t* (227) = −0.87, *p* = 0.38). The overall mean score for social appearance anxiety was 45.1 (SD = 16.62), which indicated moderate levels of anxiety in the sample.

A non‐parametric Spearman's Rho revealed a statistically significant small to medium, negative correlation between age and social appearance anxiety (*r* = −0.30, *n* = 231, *p* < 0.001), indicating that younger participants reported higher levels of social appearance anxiety. Therefore a secondary analysis of the total sample was carried out using a between‐subjects analysis of covariance examining age as a covariate. Checks were carried out to confirm homogeneity of regression and the linear relationship between the covariate and dependent variable, and these satisfied statistical assumptions. The age covariate significantly related to scores on the SAAS, (*F* (1, 228) = 41.64, *p* = 0.001). Adjusting for this covariate still resulted in a significant effect of the between‐subjects factor groups, (*F* (2, 228) = 9.91, *p* = 0.001) for level of social appearance anxiety scores across the three categories of skin severity (mild, moderate, severe). Post Hoc comparisons of the adjusted means using Tukey honestly significant difference test indicated that the mean scores in social appearance anxiety differed significantly across all three levels of severity, increasing significantly from mild to moderate and moderate to severe. Adjusted mean scores for social appearance anxiety controlling for age were: mild (*M* = 34.95, *SD* = 13.37, 95% CI [31.77, 38.12]), moderate (*M* = 44.69, *SD* = 13.32, 95 CI [42.19, 47.19]) to severe (*M* = 52.24, *SD* = 13.41, 95% CI [48.57, 55.90]), all significantly different from each other at *p* < 0.003 level.

### 4.2 Qualitative data content analysis

All 229 participants identifying as male or female provided at least one response to the four free text comment questions. Due to the unequal numbers of females (*n* = 199) and males (*n* = 30) in the sample population Table 1 presents agreed coding responses as proportions of the total sample (*n* = 229), and χ^2^ analysis was carried out for all coded variables to examine for significant differences between proportion of male and female responses. The free‐text answers were analysed in accordance with qualitative content analysis, analysing personal experiences with both inductive and deductive technique by using coping theory as the theoretical framework to inform the initial coding frame but also being open to additional themes that emerged during the analysis process. The initial coding frame created for each question allowed the identification of codes based on frequency of occurrence^38^ and two researchers independently rated the comments for content against this frame. Inter‐rater reliability across raters on all questions ranged between *κ* = 0.78 (question 4) and *κ* = 0.98 (question 1), exceeding the 0.75 level considered necessary for good inter‐rater reliability.

**TABLE 1:** Qualitative content analysis by gender

Question	Coded for	Male	Female	IRR	χ^2^
1. Challenges of living with a skin condition	Physical challenges	45.00%	53.8%	0.96	0.53
	Social challenges	51.65%	49.99%	0.87	0.00
	Constraints on behaviour	18.82%	31.14%	0.95	1.82
2. Managing challenges	Task‐coping	60.00%	59.3%	0.95	0.01
	Emotion‐coping	28.30%	28.38%	0.92	0.02
	Avoidant‐coping	18.32%	29.85%	0.91	1.19
3. How could you be better supported	Medical support	51.50%	48.72%	0.96	0.22
	Social support	3.32%	6.00%	0.97	0.03
	Stigma reduction	18.33%	24.35%	0.90	0.44
4.	Psychosocial impact	63.00%	53.00%	0.78	1.07

*Note*: IRR = inter‐rater reliability. Questions 1–3 analysed on three separate content codes independently. Question 4 coded on whether they felt there was a psychological impact. No χ^2^ analysis between males and females showed significant differences.

As shown in the χ^2^ comparison data in Table 1, there were no significant differences between the proportion of males and females' responses according to the coding frame on any of the free text comment questions. Therefore, it appears that males and females displayed similar levels of concern when responding to these questions. Due to the lack of identified gender differences, the findings below report the key themes that emerged across the sample as a whole.

### 4.3 Challenges of living with a skin condition

As shown in Table 2, the challenges of living with a skin condition were coded to three factors: *physical challenges* (reported by 49.40% of respondents) reflected issues such as dealing with physical aspects of the skin condition itself such as itchiness, flaking, soreness or pain; *social challenges* (reported by 50.80% of respondents) reflected issues around social appearance, embarrassment, stigma and misconceptions around issues as such as contagion; and *constraints on behaviour* (reported by 25.00% of respondents) reflected challenges around the choice of clothing and make‐up available due to the nature of the skin condition.

**TABLE 2:** Exemplar quotes attributed to each code (*n* = 229)

Code	Example free text comment (gender, age in years)	(%)
Challenges		
Physical challenges	‘Flaking everywhere and feeling I am making a mess. THE ITCH!!!!!’. (female, 48)	49.40%
	‘Constant itching and ripping my hair out’. (female, 31)	
	‘The itching was beyond tolerable and the resultant mess of scales and blood stains along with ruined clothing due to creams and ointments’. (male, 27).	
Social challenges	‘I have learnt to just look down at the floor and not make eye contact with people that way I don't have to look at their faces when they see my skin’. (female, 30)	50.80%
	‘People thinking it's contagious’. (female, 50).	
	‘I get self‐conscious when holding hands as its worst on my hands and wrists’. (male, 20).	
Constraints on behaviour	‘Never worn sleeveless tops because of it’. (female, 56).	25.00%
	‘Hard to put makeup on without it looking flaky and dry, skin can feel tight when rosacea flares up, general anxiety about how my skin feels/looks’. (female, 21).	
	‘Not being able to wear the clothes that I want to. Feeling like I need to cover up’. (male, 44).	
Managing the challenges		
Task‐oriented coping	‘Copious amounts of moisturiser, some steroid cream if it's particularly bad’. (male, 20).	60.00%
	‘Keep up with medication’. (female, 61).	
	‘Antidepressants’. (female, 41).	
Emotion coping	‘Stand my ground and tell myself that I am more than just my skin and what people see’. (female, 20).	28.00%
	‘I have learnt to accept my differences and see it in a positive way. Current society is a lot more accepting than it was 10 years ago now, so I feel proud to celebrate my differences as it makes me unique’. (female, 23).	
	‘Consciously trying to tell myself I am not defined by my acne or rosacea’. (female, 30).	
Avoidant coping	‘Turn a blind eye, tell myself to ignore it and that nobody can see what I can’. (female, 20).	24.10%
	‘I avoid looking in the mirror!’. (female, 58).	
	‘Cover up, avoid and push through’. (female, 44).	
Support needs		
Professional support	‘Rapid access to dermatologists when needed. Better GP training’. (male, 48)	50.10%
	‘Psychological support at diagnosis and as standard as part of treatment plan’. (female, 53).	
	‘Hope…From official, professional and easily accessed sources’. (male, 42).	
Social support	‘My family is supportive’. (female, 63).	<5.00%
	‘Support from friends and family, maybe support group’. (female, 36).	
	‘I am well supported, but…it's only part of the battle’. (female, 31).	
Stigma reduction	‘Less stigma and more realistic role models’. (female, 39).	21.30%
	‘Taking ignorance away from people. If I was treated like a normal person then life would be good’. (female, 33).	
	‘Educate people about psoriasis—you cannot catch it from me!’. (male, 63).	
Psychological impact		
	‘The real battle is happening inside of me. I can't accept myself the way I am’. (female, 21).	
	‘Having skin problems and flare ups has caused me psychological stress, depression, and isolation’. (female, 50).	
	‘I would rather miss out on opportunities than show my psoriasis in public’. (male, 45).	

### 4.4 Managing the challenges

The purpose of this question was to elucidate examples of respondents' coping strategies and resulted in three key codes that reflect the broad coping strategies identified earlier in this paper. The code of *task‐oriented coping* primarily reflected the application of topical medicine such as steroid creams and other medications and was the most endorsed theme reported by 60.00% of respondents. In comparison to the high level of endorsement of this first theme, a lower level of respondents stated additional ways in which they attempted to manage the challenges of living with a skin condition. The code *emotion‐coping* was attributed to 28.00% of respondents and encompassed coping strategies such as attempting to cope emotionally using a positive mindset or acceptance. The final identified code of *avoidant coping* was endorsed by 24.10% of respondents and reflected a range of avoidance coping strategies such as distraction or covering up visible aspects of the skin condition. This pattern of results in terms of use of coping strategies mirrors those reported in the quantitative analysis. It is important to note that many of the free text comments reflected respondents using a range of coping strategies to try and manage the multiple challenges of their skin condition and did not only use one specific coping strategy as demonstrated by one respondent:

‘Creams and distracting tasks. Covering up patches that are particularly bad and trying to forget about the patches I can't hide. Informing those around me why my skins like this to avoid questions. Keep visiting my GP and simply wait. Seek private care, however this was pricey and inaccessible again’.

### 4.5 Support needs

When participants were asked how they could be better supported in managing the challenges of their skin condition, the first most frequently identified code reported by 50.10% of the sample was *medical* *support* reflecting a desire for better support and treatments from a range of medical professionals (including GPs, dermatologists, and psychological professionals). Just over a fifth of respondents (21.30%) provided comments coded to the theme *‘stigma reduction’* and reflected a desire for less social stigma through more awareness and education of the public. Finally, the code *social* *support* (reflecting a desire for greater support from friends and family) was attributed to less than 5% of respondents suggesting most felt suitably supported by informal social support networks.

### 4.6 Psychological impact

The last free text question was written to allow the respondent autonomy to express any other opinions or personal experiences they may wish to share. However, of the 229 total respondents, 161 (70.00%) chose to provide additional comments. Whilst some of these comments expanded on previous points made, many of these clearly and succinctly articulated the wider daily psychological impact of living with a skin condition and were therefore coded to the following additional four categories (1) *mental health*, reflecting the prevalence of specific comments about distress, anxiety, depression, and low self‐esteem (2) *social comparison*, a code that reflected the significance of others in the daily lives of the respondents in both a positive and negative way (3) *adaptation* reflecting how some respondents articulated how they felt they had adjusted over time to the psychological and physical impact, and (4) *lack of recognition of psychological impact from professionals*, coded to less respondents but indicating a sense that medical professionals needed to better acknowledge the psychological impact of living with a skin condition.

## 5 DISCUSSION

The aim of this study was to gain a deeper understanding of the association between stigma, social appearance anxiety and coping strategies in males and females with skin conditions. Despite the significant response bias within our study, our overall findings suggest that males and females appear equally as emotionally affected by living with a skin condition. This is supportive of there being little difference in symptoms of depression and anxiety between men and women with acne.^26^ However, our findings differ from previous reports of females with atopic dermatitis and chronic dermatoses experiencing greater depression and anxiety symptoms,^8^, ^22^, ^23^ stigma, and social appearance anxiety,^3^ and there was little evidence for females experiencing psychiatric conditions and suicide ideation more frequently than males.^24^, ^25^ Females did appear to be more inclined to engage in avoidant coping behaviours, which has been previously speculated as resulting from the greater societal pressures placed upon female physical appearance, making avoidant coping strategies the quickest solution to evade distress.^3^, ^4^, ^39^

Despite the aforementioned limitations in the gender analyses, it is still of interest to note that were no generalisable patterns in coping or differences in levels of social appearance anxiety that can be ascribed as gender specific, suggesting that the way in which men and women cope with a skin condition may be a complex relationship between medical and demographic factors.^3^, ^9^ For example, our findings support the suggestion of younger people with chronic skin conditions being at greater risk of a negative impact from their diagnosis^18^, ^19^ and experiencing higher levels of social appearance anxiety, perhaps evidencing how the psychosocial implications of living with a skin condition may be tempered by the development of a range of coping strategies and adaptation over time.^15^ The qualitative data attained provides an insight into the specific ways in which both males and females with skin conditions attempted to adapt to daily challenges, with several respondents indicating serious mental health concerns that did not appear to be more apparent in one gender.

The negative psychological impact of living with a skin condition has been clearly documented^1^, ^3^, ^40^, ^41^, ^42^ and the present study showed that over half of all males and females felt negatively affected by social appearance, embarrassment, and self‐stigma when describing the three main challenges of living with a skin condition. The multi‐factorial challenges appeared to lead to the development of an extensive repertoire of coping strategies in both sets of analyses, reflecting the dimensions described by Lazarus and Folkman's transactional theory of stress and coping,^43^ with the most frequently reported coping style being task‐orientated, and the least reported coping style of emotion‐focussed. Participants clearly engaged with many daily problem‐focused strategies designed to tackle their skin condition, primarily around the use of topical lotions, medication or wearing loose clothing. For some, antidepressant drugs were also seen as a problem‐focused strategy, highlighting the significant psychological burden of a skin condition, and the recognition that managing stress/negative emotion is a large part of this complex biopsychosocial phenomenon.^14^

The use of emotion‐focused strategies (reported the least) appeared to reflect many younger respondents aged in their twenties and thirties trying to accept their condition and not be defined by it, suggesting a process of learning and adaptation to a chronic long‐term condition. There exists a need for more positive role models and social support from external agencies and professionals to reduce the stigma experienced by young people with skin conditions and enhance emotional coping. Evidence for avoidant coping behaviour was present in the content analysis, as respondents described coping with distractions and covering up their skin.^15^, ^20^ The reported use of maladaptive strategies such as avoiding social occasions and concealing clothing demonstrates the presence of stigma through fear of negative evaluations, and the consequential development of self‐stigma or a ‘self‐fulfilling prophecy’.^15^ However, coping theory would also claim that some strategies may reflect active distraction techniques, and therefore may be less ‘maladaptive’ and be useful in certain circumstances and to allow a ‘psychological breather.’^43^

Whilst recognising that avoidance and distraction may offer quick solutions to a potentially distressing situation (e.g., skin flare‐up) they do not resolve the problem itself. If engaged in alongside problem‐focused strategies, such as topical treatment, then it could be argued that the individual has developed an effective coping skillset. In the current study it was not the case that participants were using one strategy over another. Rather, given the prevalence of practical coping strategies in our sample and supporting the transactional theory of stress and coping^44^ it appears that some participants had developed a range of coping strategies that directly dealt with specific challenges. As such, findings were not all negative and support the notion that as greater quality of life emerges from acceptance of the existence of symptoms or altered aesthetics, a more meaningful life is fulfilled regardless of skin appearance.

It was clearly reported in this study that not only do males and females face many of the same challenges, but that the psychosocial impact of living with skin condition on individual's daily lives are significant. This was also reflected in responses regardless of perceived severity, as participants most frequently reported having moderate skin conditions, implying that psychological impairment from a skin condition is highly subjective and can affect quality of life irrespective of the degree of progression.^9^, ^10^ Whilst strategies can be developed to manage practical challenges, the social impact of living with a skin condition appeared to be so powerful that addressing stigma and public misconceptions may be vital to improving wellbeing. Given the impact of stigma on mental health^8^ and how high levels of perceived social support have been linked to better adaptation^17^, ^27^ the extent to which a lack of public understanding is real or perceived needs to be explored to increase awareness.

The study does have several limitations that must be acknowledged. There was a low response rate from males, which appears a pervasive issue as previous studies reporting on gender differences in the psychological impact of skin conditions have similarly recruited female‐dominant samples.^8^, ^22^, ^23^, ^24^, ^25^, ^26^ This may be partly explained by traditional male stereotypes around masculinity and reluctance to discuss emotions^45^, ^46^, ^47^, ^48^ and potentially exacerbated by the focus of the research; as men may have excluded themselves based on dominant gendered perceptions of the subject of appearance being ‘female’.^46^, ^48^ However, the results of this study suggest there may be little need to specifically focus on men when exploring the psychosocial impact of skin conditions, although the use of a more directed recruitment strategy targeting males would likely improve response rates. Asides from gender inequalities in response, the sample itself was self‐selecting, and participation was based on self‐reports without an empirical measure of skin severity. Along with sampling, the cross‐sectional design could limit the formulation of inferences of causation. There exists a need to investigate coping with skin conditions in this population with more robust methodology, perhaps of a longitudinal design with a more rigorous sampling.

Whilst acknowledging the limitations of this paper, the mixed methods approach to the analysis of data has yielded important insight into the psychological impact of living with a skin condition and particularly around the use of specific coping strategies to help manage the wide variety of daily challenges. Specifically, the use of a systematic approach to analysing the free text comments provided in questionnaires has provided a rich source of additional data and insight into the daily experiences and coping strategies of individuals living with skin conditions that would not have been achieved with the quantitative data alone. The need for more collaboration between medical/clinical specialists and mental health professionals to embed an all‐encompassing treatment programme into the stepped care pathway for dermatology patients has been previously noted.^2^, ^21^ Importantly, this study adds to the growing calls for additional psychosocial support for people living with skin conditions but suggests that such additional support or interventions may not need to be gender‐specific. The extent to which future support and psychological interventions have a direct and indirect effect on males, females and non‐binary individuals should continue to be measured carefully in order to identify further evidence to support this suggestion. Examples of psychosocial interventions that have previously been explored with people with skin conditions include mindfulness,^49^, ^50^, ^51^ self‐compassion, and compassion‐focussed self‐help,^52^, ^53^ and internet‐based cognitive behavioural therapy.^54^, ^55^, ^56^ However despite this, the appropriate psychological support that is clearly necessary remains lacking in certain areas.^21^ Alongside medical management of a skin condition, the individual must be fully equipped with a coping skillset to accept their diagnosis, buffer the negative impact to daily life, and learn to adjust to unpredictable flare‐ups/fluctuations in appearance.

Overall, the psychological impact of living with a skin condition and the ways in which individuals learn to cope with the social, practical, and psychological challenges appears to be similar in males and females. Whilst many deploy a range of coping strategies to manage the different demands of living with such a visibly chronic condition, there is a still a need for greater and more readily available psychological support for skin conditions. Future research could usefully acknowledge the wealth of insights provided by participants in this study, and importantly engage people living with skin conditions as co‐researchers and co‐designers.

## CONFLICT OF INTEREST

None to declare.

## AUTHOR CONTRIBUTIONS

**O. Hughes:** Conceptualization; Data curation; Formal analysis; Investigation; Methodology; Writing – original draft; Writing – review & editing. **P. B. Hutchings:** Conceptualization; Data curation; Formal analysis; Investigation; Methodology; Writing – original draft; Writing – review & editing. **C. Phelps:** Conceptualization; Data curation; Formal analysis; Investigation; Methodology; Supervision; Writing – original draft; Writing – review & editing.

## ETHICS STATEMENT

Ethical approval was obtained from the School Research Ethics Committee. Written informed consent was obtained from all patients.

## Data Availability

The data that support the findings of this study are openly available in the UWTSD research repository at https://repository.uwtsd.ac.uk.
